# Visually Induced Dizziness in Children and Validation of the Pediatric Visually Induced Dizziness Questionnaire

**DOI:** 10.3389/fneur.2017.00656

**Published:** 2017-12-05

**Authors:** Marousa Pavlou, Susan L. Whitney, Abdulaziz A. Alkathiry, Marian Huett, Linda M. Luxon, Ewa Raglan, Emma L. Godfrey, Doris-Eva Bamiou

**Affiliations:** ^1^Centre of Human and Aerospace Physiological Sciences, King’s College London, London, United Kingdom; ^2^Physical Therapy, School of Health and Rehabilitation Sciences, University of Pittsburgh, Pittsburgh, PA, United States; ^3^Physical Therapy, College of Applied Medical Sciences Majmaah University, Majmaah, Saudi Arabia; ^4^Department of Physiology, King’s College London, London, United Kingdom; ^5^Department of Audiology, Royal National Throat, Nose and Ear Hospital, University College London NHS Hospital Trust, London, United Kingdom; ^6^Audiological Medicine Department, Great Ormond Street Hospital for Children, London, United Kingdom; ^7^Division of Health and Social Care, King’s College London, London, United Kingdom; ^8^University College London Ear Institute, London, United Kingdom

**Keywords:** concussion, migraine, vestibular disorders, motion sickness, environmental dizziness triggers

## Abstract

**Aims:**

To develop and validate the Pediatric Visually Induced Dizziness Questionnaire (PVID) and quantify the presence and severity of visually induced dizziness (ViD), i.e., symptoms induced by visual motion stimuli including crowds and scrolling computer screens in children.

**Methods:**

169 healthy (female *n* = 89; recruited from mainstream schools, London, UK) and 114 children with a primary migraine, concussion, or vestibular disorder diagnosis (female *n* = 62), aged 6–17 years, were included. Children with primary migraine were recruited from mainstream schools while children with concussion or vestibular disorder were recruited from tertiary balance centers in London, UK, and Pittsburgh, PA, USA. Children completed the PVID, which assesses the frequency of dizziness and unsteadiness experienced in specific environmental situations, and Strength and Difficulties Questionnaire (SDQ), a brief behavioral screening instrument.

**Results:**

The PVID showed high internal consistency (11 items; α = 0.90). A significant between-group difference was noted with higher (i.e., worse) PVID scores for patients vs. healthy participants (*U* = 2,436.5, *z* = −10.719, *p* < 0.001); a significant difference was noted between individual patient groups [χ^2^(2) = 11.014, *p* = 0.004] but *post hoc* analysis showed no significant pairwise comparisons. The optimal cut-off score for discriminating between individuals with and without abnormal ViD levels was 0.45 out of 3 (sensitivity 83%, specificity 75%). Self-rated emotional (*U* = 2,730.0, *z* = −6.169) and hyperactivity (*U* = 3,445.0, *z* = −4.506) SDQ subscale as well as informant (*U* = 188.5, *z* = −3.916) and self-rated (*U* = 3,178.5, *z* = −5.083) total scores were significantly worse for patients compared to healthy participants (*p* < 0.001).

**Conclusion:**

ViD is common in children with a primary concussion, migraine, or vestibular diagnosis. The PVID is a valid measure for identifying the presence of ViD in children and should be used to identify and quantify these symptoms, which require specific management incorporating exposure to optokinetic stimuli.

## Introduction

Vertigo and dizziness are common but often ignored (1) symptoms in children, despite their adverse effects on psychological wellbeing, educational achievement, participation in school and leisure activity, and quality of life (QOL) (2–6). In children with concussion, dizziness at the time of injury is the strongest predictor for prolonged recovery (7). It is, therefore, imperative to identify symptoms early to make a correct diagnosis and instigate appropriate management, thus helping to avoid chronic illness (4). The most common causes of childhood vertigo and dizziness are migraine and migraine variants including vestibular migraine, peripheral vestibular syndromes, and head trauma (5, 8, 9). However, diagnosing the cause of and managing dizziness in children is often delayed or missed due to factors including a lack of awareness about the presence of these symptoms in children, knowledge on how to elicit these when taking a history by pediatric health-care providers (6, 10), and the fact that children are often unable to describe their symptoms (8, 11, 12). Questionnaires have recently been developed to address this clinical need by specifically helping children describe their dizziness (13) and its perceived impact (14) in the background of studies describing the clinical and diagnostic features of common dizziness and vertigo causes in children (9, 10, 15). Besides establishing the correct diagnosis, an important prerequisite for successful treatment of dizziness (16) is correct identification of symptom triggers. A specific constellation of dizzy symptoms that may be highly prevalent in childhood and responsive to treatment is visually induced dizziness (ViD).

Visually induced dizziness is the term used to define dizziness and/or unsteadiness symptoms triggered by a complex, distorted, large field/moving visual stimulus including the relative motion of the visual surround associated with body movement (17), due to an over-reliance on visual cues for perception and postural responses (i.e., visually dependent) (18). It is a frequent symptom associated with high disability levels, prolonged illness, and poorer clinical outcome in adults with vestibular dysfunction (19) including vestibular migraine (20). Children may be more susceptible to ViD than adults, as they rely more on visual cues for spatial orientation and their ability to integrate multisensory input to resolve sensory conflicting situations that may provoke dizziness and imbalance is not fully mature until adolescence (21–23).

Visually induced dizziness responds well to rehabilitation incorporating structured exposure to optokinetic stimulation (24, 25) and is, therefore, important to identify. Currently, there is no systematic study of ViD in childhood. This may be due to poor appreciation of ViD outside the vestibular community (17), the absence of asking children about symptom triggers (15), and lack of a validated tool that will identify and quantify ViD in children.

This study aimed to develop and validate the Pediatric Visually Induced Dizziness Questionnaire (PVID), to identify and quantify subjective ViD in children aged 6–17 years. Secondary study aims were to investigate ViD symptom severity in children with migraine, concussion, and/or vestibular disorders and to investigate the relationship between ViD and behaviors indicative of psychological problems in these children vs. healthy controls.

## Materials and Methods

### PVID Design and Validation

The PVID design and validation (26, 27) included three main phases: (i) expert panel review of initial PVID items; (ii) pilot study to assess validity and reliability of the PVID questionnaire; and (iii) main validation study and normative data collection.

#### Expert Panel Review

Three consultant pediatric audio-vestibular physicians, two physical therapists, and a psychologist constructed and selected questionnaire items from symptoms recorded in clinic reports for children diagnosed with a vestibular disorder at the Audiological Medicine Department, Great Ormond Street Hospital (GOSH), London, UK and the validated Situational Characteristic Questionnaire (SCQ) (18, 28). The SCQ measures the frequency of symptom provocation or exacerbation in environments with visual-vestibular conflict or intense visual motion (e.g., watching moving television scenes).

#### Pilot Study

The 11-item questionnaire was modified (wording alterations, bigger font size) after review for ease of completion and acceptability by 10 healthy children and 5 with a vestibular disorder aged 6–15 years. Each item was rated on a 0 (never) to 3 (most of the time) scale; a “don’t know” category was also included. The total score ranged from 0 to 33 and was normalized using the equation: total score/(total question number − “don’t know” replies) to yield a score of 0–3 with higher scores indicating greater symptom severity. For the final questionnaire, see Table 1.

**Table 1:** The pediatric visually induced dizziness questionnaire.

The following questions ask about how often you feel dizziness and unsteadiness in different places and situations. Please circle the best answer for you.				
How often in the past month have you felt the following?				
1. Riding in a car				

3	2	1	0	?
MOST OF THE TIME	SOMETIMES	ALMOST NEVER	NEVER	DON’T KNOW

2. Walking down a supermarket aisle or in a busy shop				

3	2	1	0	?
MOST OF THE TIME	SOMETIMES	ALMOST NEVER	NEVER	DON’T KNOW

3. Standing in the middle of a wide open space (e.g., large football field or square)				

3	2	1	0	?
MOST OF THE TIME	SOMETIMES	ALMOST NEVER	NEVER	DON’T KNOW

4. Watching T.V. or at the cinema				

3	2	1	0	?
MOST OF THE TIME	SOMETIMES	ALMOST NEVER	NEVER	DON’T KNOW

5. Riding on a bus				

3	2	1	0	?
MOST OF THE TIME	SOMETIMES	ALMOST NEVER	NEVER	DON’T KNOW

6. Looking at striped or moving surface (e.g., curtains, flowing water)				

3	2	1	0	?
MOST OF THE TIME	SOMETIMES	ALMOST NEVER	NEVER	DON’T KNOW

7. Using the computer (e.g., emails, computer games)				

3	2	1	0	?
MOST OF THE TIME	SOMETIMES	ALMOST NEVER	NEVER	DON’T KNOW

8. Watching moving traffic or trains (e.g., trying to cross the street or at the station)				

3	2	1	0	?
MOST OF THE TIME	SOMETIMES	ALMOST NEVER	NEVER	DON’T KNOW

9. Playing in the playground				

3	2	1	0	?
MOST OF THE TIME	SOMETIMES	ALMOST NEVER	NEVER	DON’T KNOW

10. Doing schoolwork				

3	2	1	0	?
MOST OF THE TIME	SOMETIMES	ALMOST NEVER	NEVER	DON’T KNOW

11. Participating in sports (swimming, football, basketball, dancing)				

3	2	1	0	?
MOST OF THE TIME	SOMETIMES	ALMOST NEVER	NEVER	DON’T KNOW

*Questionnaire copy not to scale*.

#### Procedure

The study comprised of completing the (a) PVID; (b) question set asking about clinically diagnosed migraines, frequent dizzy spells, severe stomach pain, vomiting, loss of consciousness, binocular vision or hearing difficulty, medication, and regular doctor visits (13); (c) Pediatric Vestibular Symptom Questionnaire (13) (PVSQ), which quantifies self-reported vestibular symptoms in children; and (d) Strengths and Difficulties Questionnaire (SDQ), a brief behavioral screening questionnaire for 4- to 17-year olds (29, 30). The SDQ informant (4–10 years) and self-report versions (11–17 years) both include five subscales: emotional, conduct, hyperactivity/inattention, peer relationship problems, and prosocial behavior. Subscale scores range between 0 and 10; total score is the sum of the first four scales (range 0–40). The English UK and US versions^1^ were used for participants in the United Kingdom and United States, respectively.

Primary school children in years 1 and 2 completed the PVID and PVSQ together with a parent or guardian at home; those in year 3 and above or in secondary school completed them independently in the classroom under the standardized direction of a research team member. General questions were completed together with a parent or guardian at home. Children with a vestibular disorder or concussion and their parents completed the questionnaires during their clinic appointment.

### Participants

Fifty-six children experiencing dizziness and/or unsteadiness due to a peripheral vestibular disorder (*n* = 14) or concussion (*n* = 42) were recruited from the Audiological Medicine Department, GOSH, and a tertiary balance center at the University of Pittsburgh Medical Center (UPMC), Pittsburgh, PA, USA, respectively. Inclusion criteria were (a) clinical diagnosis of concussion or other pathology resulting in vestibular dysfunction based on clinical history and clinical neuro-otological examination/test findings; (b) aged 6–17 years; (c) attend mainstream school. Exclusion criteria included children with (a) central nervous system involvement excluding migraine or concussion; (b) significant learning difficulties; or (c) orthopedic deficit affecting balance and gait. Diagnostic criteria are provided in Pavlou et al. (13).

Three hundred children aged 6–17 years were recruited from three primary and two secondary mainstream schools in the Greater London area. Of these,

– *n* = 58 identified on the question set as diagnosed with migraine from their primary care physician comprised the migraine group.
– *n* = 169 comprised the healthy control group.
– Children with a migraine diagnosis (*n* = 58), frequent dizzy spells (*n* = 27), vomiting, stomach pain, loss of consciousness, a neurological, psychological (*n* = 2) or orthopedic diagnosis, human immunodeficiency virus (*n* = 1), substance abuse history (*n* = 2), abnormal SDQ scores (*n* = 14) or an incomplete PVID (*n* = 1) or SDQ (*n* = 26) were excluded from the healthy group.

Written informed consent was obtained from all children and their parents before participating in the study in accordance with the Declaration of Helsinki. Ethical approval was obtained from the Institute of Child Health/GOSH Research Ethics Committee, London, UK and the institutional review board at the University of Pittsburgh, PA, USA.

### Data Analysis

IBM SPSSv.23 (IBM Corp., Armonk, NY, USA) was used for statistical analysis. Data are presented as mean (standard deviation, SD). Reliability was tested using the Cronbach alpha score for total PVID items less one item at a time to examine whether reliability decreased when an item was excluded. Exploratory factor analysis with principal axis factoring (PAF) determined construct validity. The Kaiser criterion (eigenvalue ≥1), a scree plot, and parallel analysis based on minimum rank factor analysis of polychoric correlations identified how many factors should be retained (31, 32). For the scree plot, factors lying before the point where eigenvalues began to drop were retained. The FACTOR PC program^2^ (33) was used for parallel analysis.

Receiver operating curves (ROC) assessed discriminant validity to calculate the PVID’s sensitivity and specificity in discriminating normal vs. abnormal ViD symptoms. Mann–Whitney tests determined between-group (healthy vs. patient) differences for age and questionnaire data. A Chi-Squared test determined between-group gender differences. Spearman’s correlation investigated associations between PVID, SDQ, and PVSQ scores, age, gender, binocular vision, hearing difficulty, and migraine history; only significant correlations are reported. Significant results were assumed if *p* ≤ 0.01.

Kruskal–Wallis test determined if differences existed in PVID and PVSQ scores between children with a primary migraine, concussion, or vestibular diagnosis. Pairwise comparisons were subsequently performed using Dunn’s procedure (34) with Bonferroni correction for multiple comparisons and statistical significance accepted at *p* < 0.0017.

## Results

### Demographics

Mean age significantly differed between-groups (*U* = 7,743, *z* = −2.818, *p* = 0.01). Children with a vestibular disorder reported hearing difficulty significantly more frequently than healthy children (*U* = 8,421.5, *z* = −4.148, *p* < 0.001). No between-group gender or binocular vision difficulty differences were noted. Demographic data are reported in Table 2.

**Table 2:** Participant characteristics.

Variable	Patient group (*n* = 114)	Healthy children (*n* = 169)
Age (years) (mean, range)	12.8 (6–17)	11.9 (6–17)*
**Gender (*n*)**		
Female, *n* (%)	62 (54.4%)	89 (52.7%)
Male, *n* (%)	52 (45.6%)	80 (47.3%)
Presence of migraine, *n* (%)	82 (71.9%)	–
Binocular vision difficulty, *n* (%)	23 (20.5%)	18 (10.7%)
Astigmatism, *n* (%)	2 (3.5%)	5 (3.0%)
Hearing difficulty, *n* (%)	13 (11.6%)	1 (0.6%)*
**Diagnosis, *n***		
VN (+M)	8 (2)	
BVH (+SNHL)	3 (2)	
Post-traumatic secondary hydrops (+overlap M)	1 (1)	
PVD following OM (+M)	2 (1)	
Concussion (+M)	42 (18)	
Migraine	58	

*VN, peripheral vestibular disorder, compatible with a history of past vestibular neuritis; M, meets International Headache Society diagnostic criteria for migraine; BVH, bilateral vestibular hypofunction; SNHL, sensory neural hearing loss; PVD, peripheral vestibular disorder; OM, otitis media*.

*Indicates a significant p ≤ 0.01 between-group difference between healthy children vs. the patient group comprising of all children with a primary migraine, concussion, or vestibular diagnosis.

### Internal Consistency Reliability

The PVID obtained a Cronbach alpha score of 0.90. Item-deleted statistics showed no significant change in alpha scores (range 0.88–0.90). All items correlated significantly with the total score; a corrected total-item correlation ≥0.4 suggested each item had discriminative capacity.

### Factor Analysis

The PAF’s suitability to the 11 PVID items was assessed prior to analysis. The correlation matrix revealed all variables had many correlation coefficients >0.3 and its factorability was confirmed (Kaiser-Meyer-Olkin value = 0.9, Bartlett’s Test of Sphericity *p* < 0.001) (35). Parallel analysis indicated a single factor structure and PAF analysis revealed one factor explaining 50.63% of the total variance. All items had a factor loading of >0.4 and were retained.

### Discriminant Validity and Comparison between Groups

**Figure 1:**
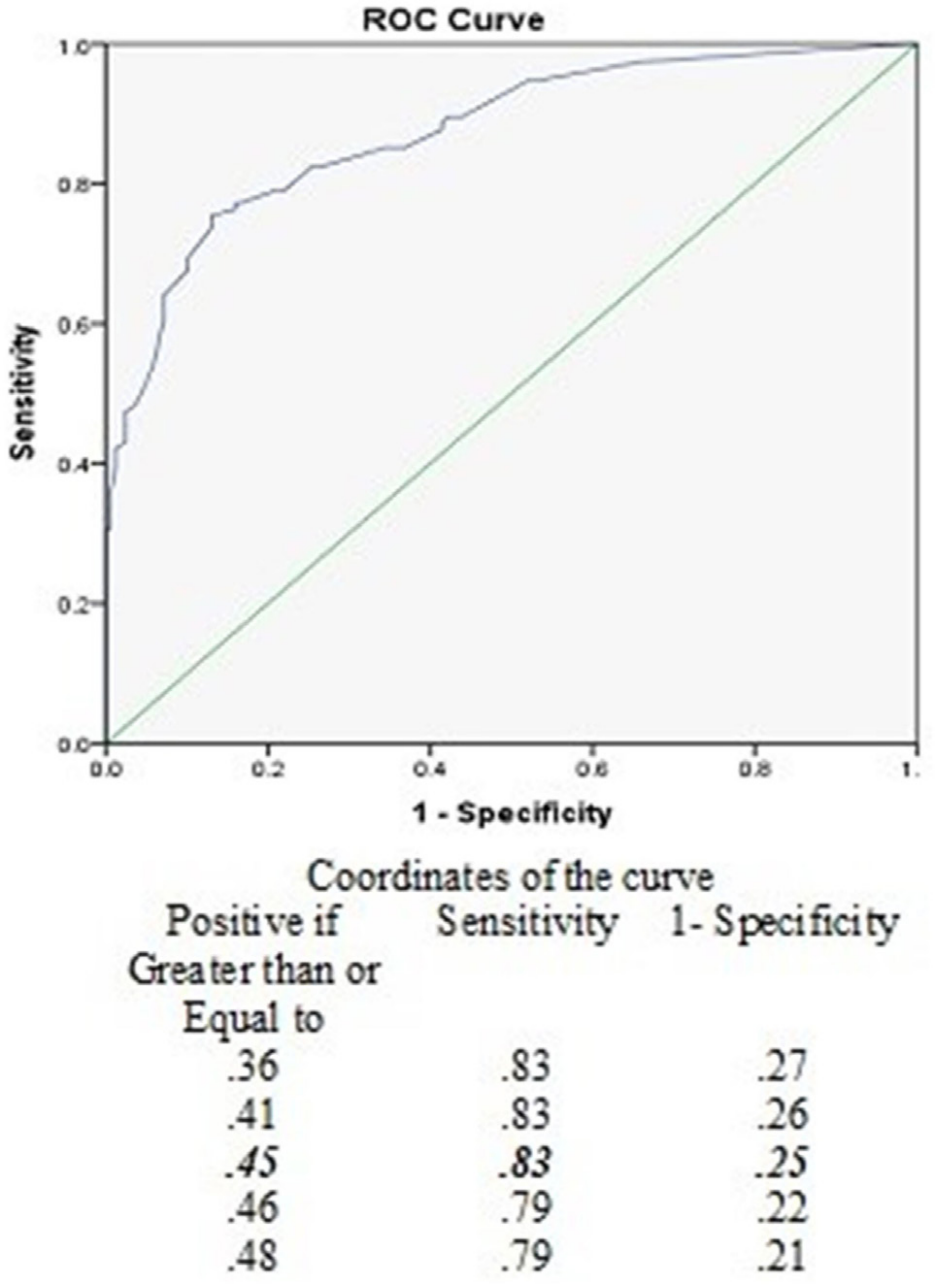
Receiver operating curves (ROC) for various cut-off levels of the pediatric visually induced dizziness questionnaire to discriminate between healthy children and the patient group.

The PVID score significantly differed between the healthy and patient group (*U* = 2,436.5, *z* = −10.719, *p* < 0.001; Table 3). ROC analysis demonstrated that the PVID could discriminate between the two groups. The optimal cut-off score was 0.45 (out of 3) with a sensitivity of 83% and specificity of 75%. Area under the curve (with 95% confidence intervals) was 0.87 (0.83–0.96, *p* < 0.001). Figure 1 shows the ROC curve with various cut-off scores for discriminating between-groups.

**Table 3:** Mean (SD) for questionnaire scores.

Questionnaires	Patient group; *n* = 114	Healthy children; *n* = 169
PVID	1.18 (0.74)	0.28 (0.33)*
PVSQ	1.35 (0.59)	0.33 (0.31)*
SDQ informant rated, *n*	*n* = 23	*n* = 45
Total score	19.86 (9.00)	10.64 (4.80)*
Emotional symptom score	4.52 (2.82)	3.02 (2.19)
Conduct problems scale	2.81 (2.40)	1.87 (1.10)
Hyperactivity scale	4.52 (2.00)	3.44 (1.98)
Peer problems score	2.57 (1.86)	2.31 (1.82)
Prosocial behavior score	8.10 (2.02)	8.84 (3.42)
SDQ self rated, n	*n* = 91	*n* = 124
Total score	13.38 (6.55)	8.89 (4.60)*
Emotional symptom score	4.26 (2.64)	2.07 (1.63)*
Conduct problems scale	2.59 (2.14)	1.81 (1.38)
Hyperactivity scale	4.62 (2.36)	3.21 (2.15)*
Peer problems score	1.91 (1.87)	1.81 (1.92)
Prosocial behavior score	7.86 (1.94)	7.15 (2.22)

*PVID, pediatric visually induced dizziness questionnaire; PVSQ, pediatric vestibular symptom questionnaire; SDQ, strengths and difficulties questionnaire scores for the informant (children <11 years old) and self-rated (≥11 years old) versions*.

*Indicates a significant p ≤ 0.01 between-group difference between healthy children vs. the patient group comprising of all children with a primary migraine, concussion, or vestibular diagnosis.

The only significant correlation noted was between PVID and PVSQ scores for both groups, whereby, higher scores for the former correlated with higher scores for the latter (healthy: *r* = 0.64, *p* < 0.001; patients: *r* = 0.43, *p* < 0.001). No correlations were noted between PVID scores and either age, hearing, or binocular vision function in either group.

### PVID and PVSQ Scores between Patient Groups

A significant between-group difference was noted for each individual patient group vs. the healthy group (vestibular: *U* = 57, *z* = −6.016, *p* < 0.001; concussion: *U* = 748.5, *z* = −8.004, *p* < 0.001; migraine: *U* = 1,631, *z* = −7.657, *p* < 0.001). As PVID score distribution was not similar for all individual patient groups, as assessed by visual inspection of a boxplot, the mean rank was used for Kruskal–Wallis analysis. Mean rank PVID scores significantly differed between patient groups [χ^2^(2) = 11.014; *p* = 0.004], whereby scores increased from a primary migraine (mean rank = 49.92) to concussion (mean rank = 59.76) to vestibular (mean rank = 82.11) diagnosis. However, *post hoc* analysis showed no significant pairwise comparisons. PVID scores were abnormal for 100, 88, and 72% of children with a primary vestibular, concussion, or migraine diagnosis, respectively.

Mean number ViD triggers per person were 7.94 (SD 2.65, range 1–11), 6.77 (SD 2.90, range 0–11), 5.27 (SD 3.31, range 0–11), and 2.05 (SD 2.34, range 0–10) in the vestibular, concussion, migraine, and healthy groups, respectively. No triggers were reported by 2 (*n* = 1), 5 (*n* = 3), and 34.3% (*n* = 58) for the concussion, migraine, and healthy groups, respectively. Percentage endorsement for each PVID item is included in Table 4.

**Table 4:** Percentage endorsement for each Pediatric Visually Induced Dizziness Questionnaire (PVID) item for the healthy and individual patient groups based on primary diagnosis.

	Endorsement for each item (%)			
PVID item	Healthy (*n* = 169)	Migraine (*n* = 58)	Concussion (*n* = 42)	Vestibular (*n* = 14)
Riding in a car	**39.9**	**60.3**	68.3	71.4
Walking down a supermarket aisle or in a busy shop	7.7	46.6	61.0	76.9
Standing in the middle of a wide open space	8.9	43.1	32.5	71.4
Watching T.V. or at the cinema	14.8	51.7	66.7	**85.7**
Riding on a bus	17.4	44.8	62.5	72.7
Looking at striped or moving surface	22.6	**60.3**	63.4	**85.7**
Using the computer	**30.2**	**62.1**	**73.8**	71.4
Watching moving traffic or trains	17.8	50.0	65.9	**85.7**
Playing in the playground	14.9	41.4	37.5	**85.7**
Doing schoolwork	**23.2**	**65.5**	**84.6**	78.6
Participating in sports	14.9	56.9	**83.9**	**92.1**

*The three highest endorsement item percentages for each group are in bold. Columns with >3 items endorsed indicate equal endorsement of certain items*.

Pediatric Vestibular Symptom Questionnaire score distribution was similar across patient groups. Median PVSQ scores showed no significant differences between patient groups. 100, 97.6, and 86.2% of children with a primary vestibular, concussion, or migraine diagnosis, respectively, had abnormal PVSQ scores (13).

### Strength and Difficulties Questionnaire

Significant between-group differences were noted for the self-rated SDQ with significantly higher scores for the patient vs. healthy group for the emotional (*U* = 2,730.0, *z* = −6.169, *p* < 0.001), and hyperactivity (*U* = 3,445.0, *z* = −4.506, *p* < 0.001) subscales and total score (*U* = 3,178.5, *z* = −5.083, *p* < 0.001). Informant-rated SDQ scores showed a significant between-group difference for the total score only (*U* = 188.5, *z* = −3.916, *p* < 0.001). Mean self- and informant-rated SDQ scores were within normal ranges for healthy children, as expected based on exclusion criteria, and for older children in the patient group (age ≥11 years old, completed self-rated SDQ), who were not excluded if they had borderline (*n* = 11) or abnormal scores (*n* = 8) (Table 3). For younger children in the patient group (informant-rated SDQs), mean total and emotional subscale scores were abnormal, conduct, and peer problem scores borderline while hyperactivity and prosocial behavior scores were within normal ranges (29, 36).

In the healthy group, a significant relationship was noted between mean self-rated SDQ emotional and both PVID (*r* = 0.30, *p* < 0.001) and PVSQ (*r* = 0.24, *p* = 0.007) scores and between the PVSQ with both hyperactivity (*r* = 0.27, *p* = 0.002) and total SDQ (*r* = 0.25, *p* = 0.006) scores, whereby higher SDQ scores correlated with higher questionnaire scores. In the patient group, no significant correlations were noted for the self-rated SDQ; however, for informant-rated SDQs, a significant relationship was noted between total and emotional subscale scores with PVID (total: *r* = 0.65, *p* = 0.001; emotional: *r* = 0.69, *p* = 0.001), PVSQ (total: *r* = 0.60, *p* = 0.004; emotional: *r* = 0.55, *p* = 0.01), and migraine (total: *r* = 0.74, *p* = 0.01; emotional: *r* = 0.55, *p* = 0.01), whereby higher SDQ scores were associated with higher questionnaire scores and migraine history.

## Discussion

This is the first study to provide evidence that ViD exists in childhood, with significant differences noted between the healthy and patient group for ViD symptom severity, number of PVID items endorsed, and SDQ scores. The number of children reporting visual environmental triggers, mean number of items endorsed by each child in the patient groups, and percentage endorsement for each item is significantly greater than that noted in adults with a vestibular disorder (37). These findings may partly be due to the types of environmental triggers included in each scale, but are mostly likely explained by factors specific to children including immaturity in motion processing systems (38) and a prolonged time course for development of the ability to integrate multisensory cues to resolve sensory conflicting situations (23), with increased reliance on visual cues for spatial orientation (22). Children show less selective or robust responses to optic flows differing in pattern or speed, and motion processing networks continue to experience “fine-tuning” throughout the transition to adulthood (38). It is interesting thus to note that “standing in a wide-open space” is one of the items least endorsed for children with concussion and/or migraine, suggesting less susceptibility to environments with insufficient visual information and greater susceptibility to visual information overload (striped/moving surfaces, doing schoolwork, i.e., flickering effect when eye movements pass over print, sports, e.g., number and tracking of team players).

Children with a vestibular disorder endorsed all PVID items highly. The main triggers endorsed by children with concussion and/or migraine were also diverse, with computer use and doing schoolwork identified as key triggers. Recent studies have indicated an association between increased screen time, headache frequency (39), dizziness symptoms (39, 40), and psychological distress (41) in children. Time spent on smartphones, tablets, and computers should be considered when asking children about their symptoms and symptom triggers.

Visually induced dizziness is believed to be due to visual dependency (42) and is supported by studies showing increased visual cortical excitability in persons with vestibular dysfunction (43) and migraineurs (44). Similarly, in persons with concussion, an increased coherent motion threshold has been noted, indicating damage to the magnocellular pathway, which is directly involved in visual motion processing (45). The detection of coherent motion is required to be able to detect the correct direction of movement. Therefore, it follows that an increase threshold indicates increase visual motion sensitivity. These findings are consistent with visual motion sensitivity and vertigo symptoms reported by persons with concussion in their natural environments (45).

The relationship between motion sickness and increased visual dependence also exists with a stronger influence of disorienting visual stimuli on verticality perception (46) and an inability to accurately use available visual input to resolve conflicting sensory information (47). The most common symptom triggers in healthy children were those specifically relating to motion sickness, including computer use, with the percentage similar to that noted in previous work (48). All healthy individuals may become motion sick, but individual susceptibility varies widely and is primarily due to genetic hereditability (49) and gender, with girls more susceptible than boys (48). No age or gender effect was noted on PVID scores for any group. This may be because correlation analysis assessed the relationship between mean PVID scores with age and gender, rather than individual items relating to motion sickness.

No relationship was noted between the presence of a binocular visual abnormality and PVID scores. This finding is in agreement with adult data showing no difference in baseline ViD scores between persons with a vestibular disorder with and without binocular vision abnormalities (50). This does not exclude though a potential contribution of the visual system to the presence of these symptoms, although the term ViD specifically refers to the environmental triggers of these symptoms.

Children with a primary migraine diagnosis had been diagnosed by their primary care physician. An abnormal PVSQ score was noted in 86.2%, indicating increased vestibular symptoms, while PVID scores were abnormal for 72% with 95% endorsing at least one symptom trigger. Vestibular symptoms of dizziness and ViD may indicate vestibular migraine (20), which may occur at any age and is widely underdiagnosed (51, 52). In one study, vestibular migraine accounted for 20.2% of the diagnoses, but was suspected by the referring health-care professional in only 1.8% (53). Dizziness/vertigo are common symptoms in children with migraine (54) and require a comprehensive neuro-otological assessment for correct diagnosis and exclusion of other diagnoses that may cause these symptoms (20). However, none of the children in the migraine group had been referred for a neuro-otological assessment.

Children with concussion, migraine, and/or vestibular disorder should also be screened for associated psychological symptoms, perceived handicap, and QOL (55–58). Mean SDQ scores were increased compared to healthy controls for both self- and informant-rated versions. Significant correlations were noted between questionnaires, migraine history, and SDQ for the informant-rated version in patients, consistent with adult studies (59, 60), indicating a contributory role of the limbic system for these symptoms (61). Parent SDQ reports are more discriminating than youth self-reports (62), thus these findings may more accurately represent the true associations between these measures compared to self-report data.

Increased sensitivity to visual motion stimuli has been reported in persons with persistent postural-perceptual dizziness (PPPD), functional dizziness, and chronic subjective dizziness (CSD) (63–65). Children with a vestibular disorder or concussion had not received any of these diagnoses, and there are no studies to our knowledge that have evaluated the presence of these conditions in a pediatric population. It is unknown if any children in the migraine group may have been provided with an additional diagnosis of PPPD, CSD, or functional dizziness if they had received a full neuro-otological assessment. However, our data show that increased ViD symptoms are common in children with a vestibular disorder, migraine, and/or concussion, and it has been argued that persons with ViD should receive specific rehabilitation regardless of what gives rise to this symptoms’ constellation (21).

Greater difficulties at school and decreased academic performance are reported for children with concussion or migraine, with contributing factors including headache severity and impact, work accumulation from missing school, potential stress associated with this, and cognitive effects particularly following concussion (57, 66). Our data suggest that increased ViD symptoms may also be a contributing factor to decreased school functioning and should be considered.

A significant relationship was also noted between PVID, PVSQ, and self-rated SDQ scores in healthy participants. In healthy adolescents, a significant correlation exists between subjective health complaints (i.e., dizziness, fatigue), focusing on re-occurring symptom frequency rather than diagnoses, and higher SDQ scores, indicating greater emotional/behavioral problems (67). We hypothesize that in, healthy participants, the relationship between PVSQ, PVID, and SDQ scores may result from the presence of subjective health complaints in a percentage of participants.

The PVID provides a valid tool that may reliably identify ViD. A ROC area under the curve indicates a test with “good accuracy” in separating those with and without abnormal ViD symptom levels (68). Construct validity was shown by factor analysis, which retained all items organized into one dimension (i.e., single factor structure) representing ViD symptoms. Using two rules to determine the number of dimensions in the data adds robustness to the decision and subsequent data interpretation (69). In the current study, the Kaiser criteria and scree plot were supplemented by a parallel analysis with minimum rank factor analysis. Parallel analysis, which also extracted one dimension, is the only approach that formally tests the probability that a factor is due to chance and allows for a high degree of confidence regarding the number of factors to extract (70). Cronbach’s alpha above the recommended values of 0.8 (71) or 0.7 for a new instrument (72, 73) indicated high internal consistency and a ≥0.4 correlation for all items with the total score also suggested that all items contribute to the PVID’s reliability. The PVID may thus be a clinically valuable tool to assess children with dizziness-related symptoms to inform diagnosis and appropriate management.

Some study limitations were present. No neuro-otological assessment was completed for children with a primary migraine diagnosis as they were recruited from mainstream schools and no information was collected about migraine characteristics, academic performance, and QOL, which may have provided greater insight into the relationships between these factors and ViD. The absence of data regarding wearing glasses and further details of squints and stereoscopy is also a study limitation. Further research on test–retest reliability and responsiveness to change over time is needed to further validate the PVID for clinical use. Currently, the questionnaire is not validated for children with learning disabilities, as questionnaires should be specifically adapted for populations experiencing reading and comprehension difficulties (74). This should be considered particularly when used in children with concussion who may experience cognitive deficits (75).

### Conclusion

Increased ViD symptoms are present in children with migraine, concussion, and/or vestibular disorders. Increased knowledge translation is required so that health-care professionals are aware of these symptoms and how to identify them. The PVID is a reliable and valid measure for assessing ViD presence and severity in children. A better understanding of the relationships between symptoms, symptom triggers, functional domains, and QOL may improve the management approach and outcome for these children.

## Ethics Statement

This study was carried out in accordance with the recommendations of the Institute of Child Health/GOSH Research Ethics Committee, London, UK and the institutional review board at the University of Pittsburgh, PA, USA. Ethical approval was obtained from the Institute of Child Health/GOSH Research Ethics Committee, London, UK and the institutional review board at the University of Pittsburgh, PA, USA. Written informed consent was obtained from all children and their parents before participating in the study in accordance with the Declaration of Helsinki.

## Author Contributions

MP conceptualized and designed the study, contributed to the design of the data collection instrument, collected data for children with vestibular disorders from GOSH, London, UK, contributed to data analyses, drafted the initial manuscript, and approved the final manuscript as submitted. SW contributed to the design of the data collection instrument, coordinated and supervised data collection at UPMC, Pittsburgh, PA, USA, critically reviewed and revised the manuscript, and approved the final manuscript as submitted. AA was involved in data collection at UPMC and carried out the initial analyses. MH coordinated and supervised data collection at all five sites for the healthy control group, critically reviewed and revised the manuscript, and approved the final manuscript as submitted. EG contributed to the study design, critically reviewed and revised the manuscript, and approved the final manuscript as submitted. LL, ER, and DEB contributed to the design of the data collection instrument, recruited children with vestibular disorders from GOSH, critically reviewed and revised the manuscript and approved the final manuscript as submitted. DEB also contributed to data analyses.

## Conflict of Interest Statement

The authors declare that the research was conducted in the absence of any commercial or financial relationships that could be construed as a potential conflict of interest.

## Footnotes

^1^www.sdqinfo.org.

^2^http://psico.fcep.urv.es/utilitats/factor.

## Abbreviations

PVID, pediatric visually induced dizziness questionnaire; ViD, visually induced dizziness; QOL, quality of life; GOSH, Great Ormond Street Hospital; UPMC, University of Pittsburgh Medical Center; SDQ, strength and difficulties questionnaire; PVSQ, pediatric vestibular symptom questionnaire; PAF, principal axis factoring; ROC, receiver operating curves; PPPD, persistent postural-perceptual dizziness; CSD, chronic subjective dizziness; SD, standard deviation.
